# Interleukin-37 mediates the anti-oral tumor activity in oral cancer through STAT3

**DOI:** 10.1515/med-2025-1173

**Published:** 2025-05-26

**Authors:** Jing Fang, Kunshan Li, Liyuan Zhang, Ying Zhang, Yifan Wang, Jing Zhang

**Affiliations:** Endoscopy Clinic, Qinhuangdao Hospital of Traditional Chinese Medicine, Qinhuangdao, Hebei, 066000, China; Department of Stomatology, The Fourth Hospital of Hebei Medical University, Shijiazhuang, Hebei, 050017, China; Department of Stomatology, Qinhuangdao Hospital of Traditional Chinese Medicine, Qinhuangdao, Hebei, 066000, China; Department of Stomatology, The Third Hospital of Shijiazhuang City, Shijiazhuang, Hebei, 050011, China; Department of Stomatology, Affiliated Hospital of Hebei University, Baoding, Hebei, 071000, China; Department of Medical Periodical Press, The Fourth Hospital of Hebei Medical University, 12 Health Road, Shijiazhuang, Hebei, 050017, China

**Keywords:** IL-37, oral cancer, cell growth, STAT3

## Abstract

Oral cancer constitutes a significant public health challenge, and gaining insights into the pathogenesis of oral cancer is crucial for the development of innovative therapeutic approaches. To address this objective, this study investigates the impact of interleukin-37 (IL-37) on oral cancer and its underlying mechanisms. Two oral cancer cell lines, HN13 and HSC-6, were employed in the study. Our findings reveal that IL-37 markedly inhibits cell viability and induces apoptosis in oral cancer cells. IL-37 attenuates the proliferation of oral cancer cells induced by lipopolysaccharide and tumor necrosis factor-alpha, while knockdown of *IL-37* exacerbates this induction. Furthermore, IL-37 demonstrates anti-inflammatory effects on oral cancer cells. The modulation of inflammation, proliferation, apoptosis, and migration by IL-37 is mediated through the STAT3 pathway. The outcomes of this study contribute valuable insights to a deeper understanding of oral cancer pathogenesis and pave the way for the development of novel drugs for the treatment of this disease.

## 1 Introduction

Oral cancer constitutes a significant public health challenge, with a growing incidence observed among young men and women [1]. The risk of developing oral cancer rises with age, and the majority of cases are reported in individuals aged 50 years and above [1]. Despite advancements in medical care, the 5-year survival rate for oral cancer patients has not witnessed substantial improvement over the past decades, lingering at around 50% [2]. Geographical variations in incidence are notable, with Papua New Guinea identified as having the world’s highest rate of oral cancer [2]. The etiology of oral cancer is multifactorial, with factors such as tobacco use, smoking, smokeless tobacco (snuff or chewing tobacco), alcohol consumption and areca nut intake, excessive sunlight exposure, reverse end smoking, and human papilloma virus being implicated [3]. The management of oral cancers is intricate, given the complex functional and aesthetic considerations associated with treating tumors in this region [4]. Prioritizing early diagnosis of oral cancer and exploring emerging strategies for targeted treatment emerge as effective approaches to reduce mortality and enhance the quality of life for patients [4]. An in-depth understanding of the pathogenesis of oral cancer is pivotal in the development of innovative therapies for this particular cancer type.

As a newly identified member of the IL-1 family, interleukin‐37 (*IL‐37*) possesses the ability to counteract the pro‐inflammatory effects of *IL‐18* (Figure S1). It achieves this by competing for the IL‐18 receptor (specifically the IL‐18Rα subunit) and dampening MyD88 activity when binding to the Ig‐like Toll/IL‐1R (TIR) receptor known as *TIR8* [5]. IL-37 has been proven to inhibit both systemic and local inflammation by diminishing the levels of pro-inflammatory mediators [6]. In a murine viral myocarditis model induced by coxsackievirus B3, IL-37 led to an increase in the survival rate and body weight, while concurrently suppressing the production of *IL-6* and *IL-17A* [7]. The IL-37 receptor comprises two distinct subunits: the IL-18 receptor α chain (*IL-18Rα*) and single immunoglobulin IL-1 receptor-related protein (*SIGIRR*). Signaling through IL-37/IL-37 receptor activates multiple intracellular switches, leading to the down-regulation of proinflammatory genes and the suppression of cytokine production [5]. IL-37 has been implicated in the modulation of infectious diseases. Notably, acute HBV infection has been associated with the down-regulation of IL-37, potentially linked to enhanced CD8^+^ T cell cytotoxicity and liver damage [8]. Variants of the *IL-37* gene, such as rs3811046 and rs3811047, have been suggested to be associated with susceptibility to COVID-19 in the Iraqi population [9]. Accumulating evidence points to the intricate role of IL-37 in regulating the pathogenesis of oral cancers. Elevated expression levels of IL-37 have been observed in lung adenocarcinoma (LUAD) tumors, and the expression profiles of both IL-37 and its receptor *SIGIRR* are correlated with LUAD development and tumor stage [10]. Additionally, IL-37 has been found to exert anti-tumor immunity by indirectly promoting dendritic cell recruitment and activation in hepatocellular carcinoma (HCC) [11]. Given these findings, it is compelling to investigate the effects of IL-37 on oral cancer.

The signal transducer and activator of transcription (*STAT*) proteins constitute a family of cytoplasmic transcription factors characterized by an overall modular structure with functional domains [12]. Among these, *STAT3* is a member of the *STAT* family, operating as a cytoplasmic transcription factor that facilitates signal transduction from the plasma membrane to the nucleus in diverse cell types [12]. Engaging in various biological processes such as cell proliferation, survival, differentiation, and angiogenesis, *STAT3* is pivotal for normal cellular functions [12]. In non-cancerous cells, *STAT3* undergoes transient activation, predominantly through phosphorylation, to relay transcriptional signals from cytokines and growth factor receptors at the plasma membrane to the nucleus [13]. In the context of inflammation, *STAT3* is activated by the IL-6-type cytokine family, encompassing *IL-6*, *IL-11*, *IL-22*, *IL-27*, *IL-31*, oncostatin M, cardiotrophin 1, ciliary neurotrophic factor, cardiotrophin-like cytokine factor 1, and leukemia inhibitory factor [13]. Hyperactivation of *STAT3* is commonly observed in human cancers and is generally associated with unfavorable clinical prognosis [14]. Widely implicated in tumorigenesis, *STAT3*, when activated, upregulates the mRNA levels of numerous genes involved in cell growth and apoptosis. These include cyclins D1, D2, D3, A, and B, *Cdc25A*, *Cdc2*, *c-Myc*, *PLK1*, *Pim-1/2*, *Cten*, *survivin*, *Bcl-xL*, *IAPs*, and *Mcl-1*. The coordinated action of these upregulated genes contributes to the oncogenic transformation of cells [15]. Moreover, *STAT3* plays a crucial role in the G1 to S phase cell cycle transition by modulating the expression of cyclins D1, D2, D3, A, and *Cdc25A*, while concurrently regulating *p21* and *p27*, thereby influencing cancer-related activities [16]. Given its multifaceted involvement in cellular processes, exploring the role of *STAT3* in the regulation of oral cancers becomes particularly intriguing.

The study employed two oral cancer cell lines, namely HN13 and HSC-6, to systematically investigate the impact of *IL-37* on the proliferation and apoptosis of oral cancer cells. The research delved into the intricate mechanisms through which *IL-37* regulates oral cancers. The outcomes of this study hold significant importance for advancing our understanding of the pathogenesis of oral cancers and pave the way for the development of targeted therapies for the treatment of this disease.

## 2 Materials and methods

### 2.1 Reagents

Recombinant human IL-37 was procured from PeproTech (catalog number: 200-39). Lipopolysaccharide (LPS) was obtained from Beyotime (Beyotime, Shanghai, China, catalog number: ST1470-10mg), while tumor necrosis factor-alpha (TNF-α) was sourced from Sigma (Sigma, catalog number: H8916-10UG). Stattic, a specific STAT3 inhibitor, was purchased from Sigma (Sigma, catalog number: 573099).

### 2.2 Cell lines

Normal oral keratinocytes (*NOK*), oral squamous cell carcinoma cell lines, including *HN-13* and *HSC-4*, were obtained from the bio-resource center of Hebei Medical University. *NOK*, *HN-13*, and *HSC-4* cells were cultured in Dulbecco’s Modified Eagle Medium (DMEM) (ThermoFisher, catalog number: 10569010), supplemented with 10% FBS (ThermoFisher, catalog number: 16140071) and 1% penicillin–streptomycin (ThermoFisher, catalog number: 15140148). All cells were maintained at 37°C in a humidified chamber containing 5% CO_2_.

### 2.3 Real-time quantitative PCR (qPCR)

Total RNA was extracted from *HN-13* and *HSC-4* cells using TRIzol reagent (Invitrogen, Carlsbad, CA, USA, catalog number: 15596026). RNA quantification was performed using Qubit 4 (ThermoFisher). cDNA was synthesized from RNA using the Takara 6210A PrimeScript™ II 1st Strand cDNA Synthesis Kit (Takara). The expression levels of mRNAs were evaluated using the SYBR Green Master Mix (Invitrogen, catalog number: A46112) in an ABI Prism 7900HT Sequence Detection System (Life Technologies, Carlsbad, CA, USA). qPCR was carried out at 50°C for 2 min and 95°C for 2 min, followed by 40 cycles at 95°C for 15 s, 60°C for 1 min, and an extension at 72°C for 1 min, with a final extension step at 72°C for 10 min. *GAPDH* served as an internal control. The specific primers used are listed in Table 1. Data were calculated and analyzed using the comparative threshold cycle (2^−∆∆Ct^) method [17]. Expression of *IL-37* in the three cell lines is indicated in Figure S2.

**Table 1:** Primers used in the present study

Gene name	Primer	Sequence
Caspase3	Forward	GGCGGTTGTAGAAGAGTTTCG
	Reverse	TCACGGCCTGGGATTTCAAG
IL23	Forward	TGCCAGCAGCTTTCACAGAA
	Reverse	TTGCAAGCAGAACTGACTGT
IL1b	Forward	CCAAACCTCTTCGAGGCACA
	Reverse	GCTGCTTCAGACACTTGAGC
IL6	Forward	CCGGGAACGAAAGAGAAGCTC
	Reverse	ACCGAAGGCGCTTGTGGAG
IL17	Forward	CACCTTGGAATCTCCACCGC
	Reverse	GGATCTCTTGCTGGATGGGG
IL-37	Forward	CAAGCCTCCCCACCATGAAT
	Reverse	GCAAAGAAGATCTCTGGGCG
Ki67	Forward	GGATCGTCCCAGTGGAAGAG
	Reverse	CAAACAAGCAGGTGCTGAGG
GAPDH	Forward	AATGGGCAGCCGTTAGGAAA
	Reverse	GCCCAATACGACCAAATCAGAG

### 2.4 Flow cytometric analysis of Annexin V/propidium iodide (PI) staining

*HN-13* and *HSC-4* cells were seeded in six-well plates (Costar; Corning, Inc.; 150,000 cells/well). Upon reaching 60–70% confluence, cells were subjected to the relevant treatments in a cell culture incubator (37°C, 5% CO_2_). After treatment, cells were harvested with trypsin/EDTA and stained for 15 min at room temperature using the FITC Annexin V Apoptosis Detection Kit I (cat. no. 556547; BD Pharmingen). Flow cytometry analysis was performed using a FACSCanto II (BD Biosciences). In Section 3, PI + Quadrants Q1 and Q2, respectively, represent necrosis and late-stage apoptosis/secondary necrosis, Quadrant Q4 represents viability (AnnV−/PI−), and Quadrant Q3 (AnnV+/PI−) represents early-stage apoptosis.

### 2.5 Transwell migration experiment

Transwell chambers (24-well, 8.0 μm pore membranes, Corning USA) were employed following the manufacturer’s protocol. Briefly, before seeding cells, 100 μL of 1:8 DMEM-diluted Matrigel (BD, USA) was added to each well and incubated at 37°C for 6 h before cell seeding onto the membrane. Subsequently, 2 × 10^4^ *HN-13* and *HSC-4* cells per well were seeded in the upper chamber in 100 μL of serum-free medium, while 600 μL of complete medium was added to the lower chamber as a chemoattractant. After incubation for 24 h at 37°C, the cells remaining on the upper surface of the membrane were removed with cotton swabs, and the cells on the lower surface of the membrane were considered migrated cells. Following fixation with 4% paraformaldehyde and staining with 0.1% crystal violet solution, the cells that passed through the filter were photographed using an inverted fluorescence microscope.

### 2.6 Measurement of cytotoxicity using cell counting kit-8 (CCK8) assay

CCK-8 (cat no. C0037; Beyotime Institute of Biotechnology) was employed to assess cytotoxicity in both cell lines, following the manufacturer’s instructions. Briefly, *HN-13* and *HSC-4* cells were seeded into 96-well plates at a cell density of 5 × 10^4^ cells/mL overnight. After 24 h, the cell culture medium was replaced with indicated concentrations of chemicals, and the treatment continued for 48 h. CCK-8 solution (0.5 mg/mL; 100 μL) was added to each well and incubated for 3 h at 37°C, followed by the detection of optical density values at 450 nm using an Infinite M200 PRO Multimode Microplate Reader (Tecan Group, Ltd). The percentage of live cells was calculated relative to the control.

### 2.7 Small interfering (si)RNA-based knockdown (KD) assay

Gene KD was achieved through siRNA technology. The siRNA sequences targeting mammalian target of rapamycin (mTOR) were designed using siRNA-Target-Finder (GeneScript, https://www.genscript.com/tools/sirna-target-finder? page_no = 1&position_no = 2&sensors = googlesearch), synthesized, and procured from Synbio Technologies. The sequence of the negative control siRNA in the empty vector was 5′-UUCUCCGAACGUGUCACGU-3′, and the sequence of siRNA-IL-37 was 5′-AAGTACTGGTCCTGGACTCTG-3′. The siRNAs (non-targeting control siRNA and target siRNA) were transiently transfected into the *HN-13* and *HSC-4* cell lines using FuGENE HD Transfection Reagent (cat. no. E2311; Promega Corporation) following the manufacturer’s instructions in the cell culture incubator (37°C, 5% CO_2_). Transfection with siRNA occurred 24 h before subsequent experiments, and the KD efficiency was assessed using RT-qPCR and western blot assays following the protocols described in this study.

### 2.8 Statistical analysis

All data were expressed as mean ± standard error of the mean. For statistical analysis of continuous variables, one-way ANOVA and Tukey’s *post-hoc* test were applied. Categorical variables were analyzed using Fisher’s exact tests. Correlation analysis (Pearson) and statistical computations were carried out with GraphPad Prism 5.0 software (GraphPad Software, Inc.). A *p*-value of less than 0.05 was considered statistically significant.

## 3 Results

### 3.1 IL-37 significantly suppressed growth and increased apoptosis of oral cancer cells

**Figure 1:**
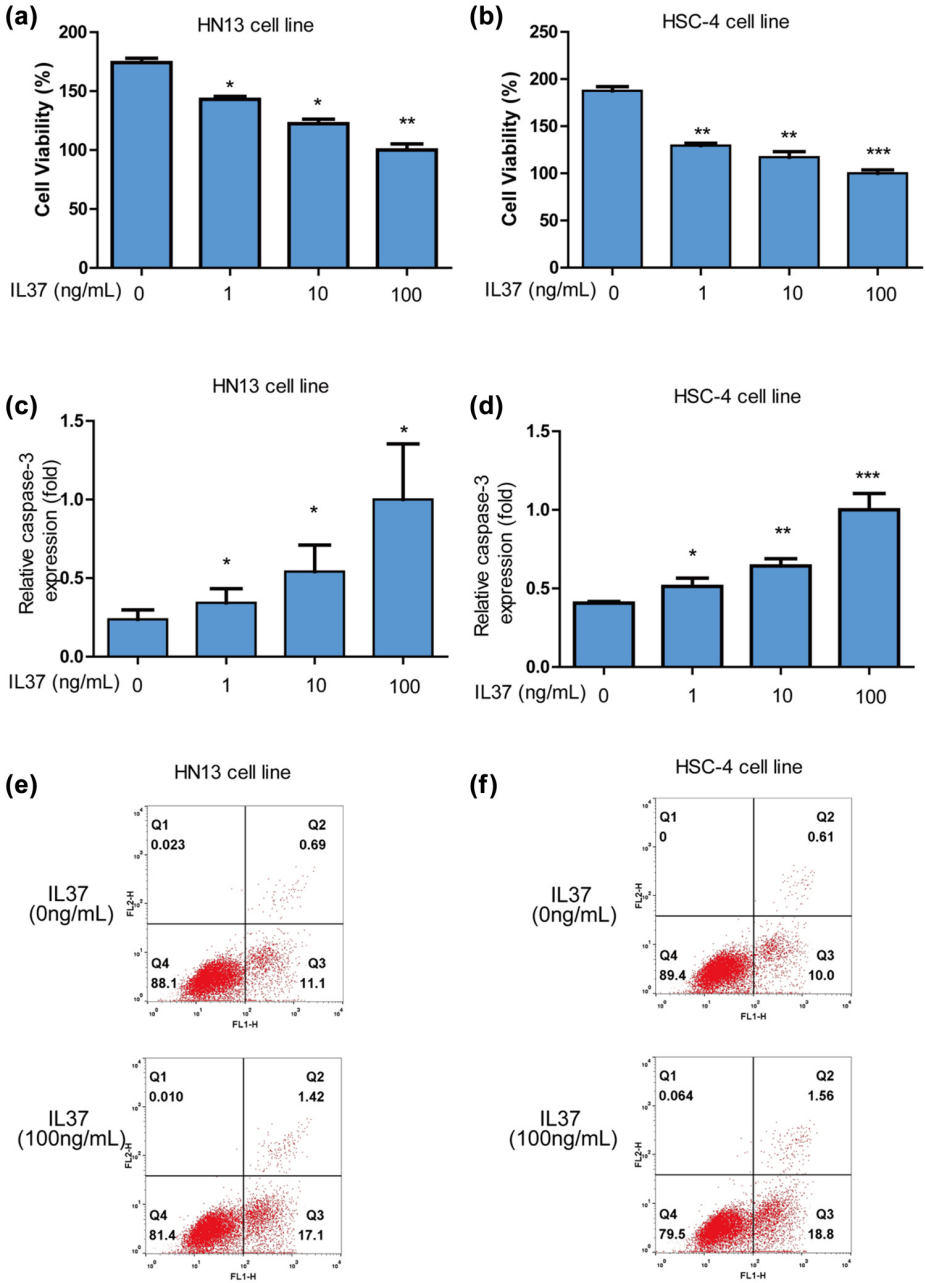
*IL-37* significantly suppressed growth and increased apoptosis of oral cancer cells. (a) *IL-37* significantly decreased cell viability of HN13 oral cells in a dose-dependent manner. (b) *IL-37* significantly decreased cell viability of HSC-4 oral cells in a dose-dependent manner. (c) *IL-37* significantly increased apoptosis of HN13 oral cells in a dose-dependent manner. (d) *IL-37* significantly increased apoptosis of HSC-4 oral cells in a dose-dependent manner. (e) *IL-37* (100 nM) significantly increased apoptosis of HN13. (f) *IL-37* (100 nM) significantly increased apoptosis of HSC-4 cells in a dose-dependent manner.

To investigate the impact of IL-37 on the activity of oral cancer cells, HN13 cells were subjected to varying doses (0, 1, 10, 100 nM) of IL-37. The results indicated a significant dose-dependent decrease in cell viability in HN13 oral cells treated with IL-37 (Figure 1a). Similarly, IL-37 exhibited a dose-dependent reduction in cell viability for HSC-4 oral cells (Figure 1b). Notably, IL-37 induced a significant dose-dependent increase in apoptosis for HN13 oral cells (Figure 1c), and a similar effect was observed for HSC-4 oral cells (Figure 1d). To further validate the impact of IL-37 on oral cancer cells, fluorescence-activated cell sorting analysis was performed. The results revealed that IL-37 (100 nM) significantly increased apoptosis in both HN13 (Figure 1e) and HSC-4 (Figure 1f) oral cancer cells. In summary, these findings demonstrate that IL-37 effectively inhibits growth and promotes apoptosis in oral cancer cells.

### 3.2 IL-37 alleviated LPS and TNF-α-induced proliferation of oral cancer cells

**Figure 2:**
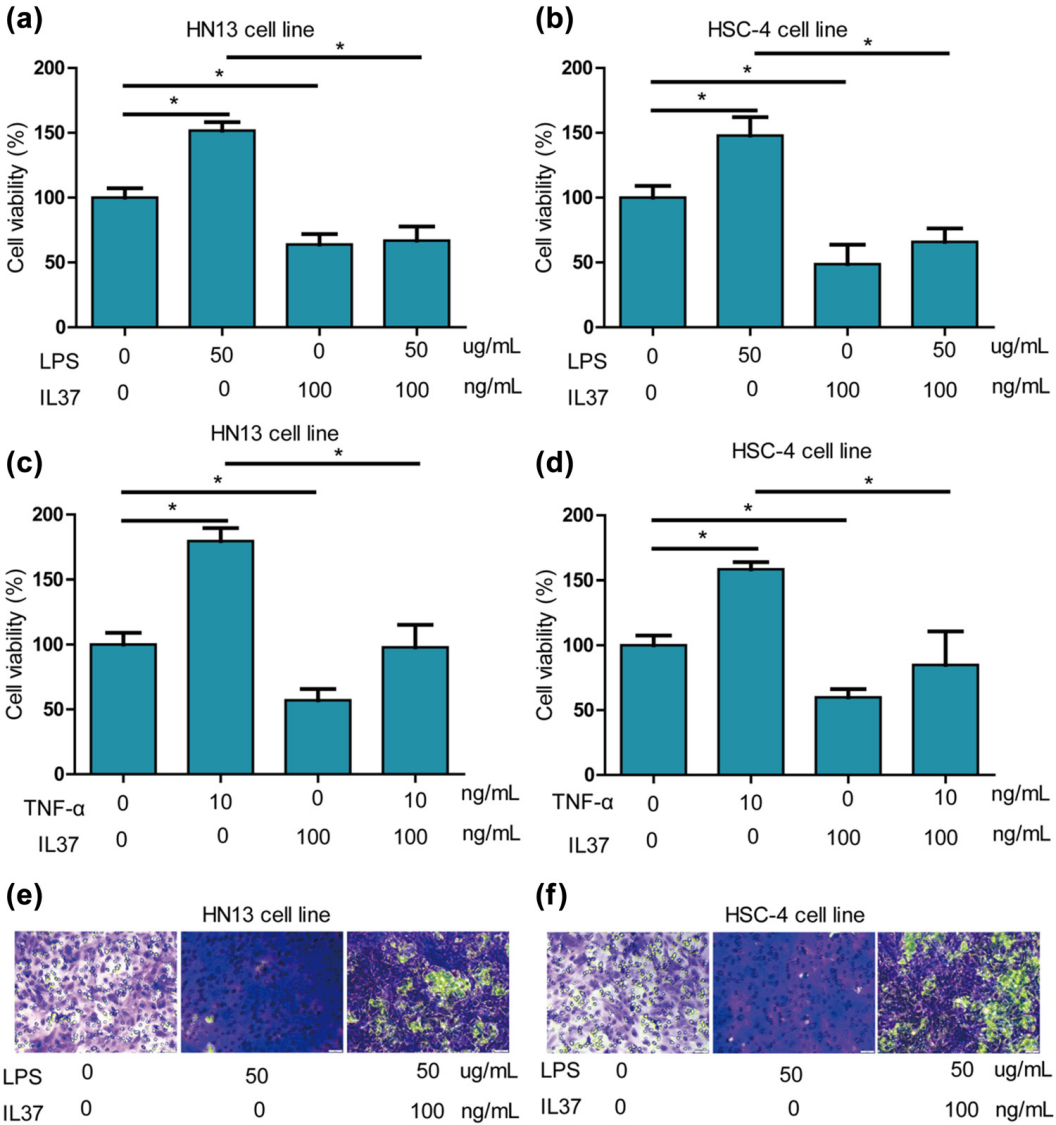
*IL-37* alleviated LPS and TNF-α-induced proliferation of oral cancer cells. (a) LPS significantly increased cell viability of HN13 cells, and *IL-37* significantly alleviated LPS-induced increase of cell viability of HN13 cells. (b) LPS significantly increased cell viability of HSC-4 cells, and *IL-37* significantly alleviated LPS-induced increase of cell viability of HSC-4 cells. (c) TNF-α (20 ng/mL) significantly increased cell viability of HN13 cells, and IL-37 significantly alleviated TNF-α (20 ng/mL)-induced increase of cell viability of HN13 cells. (d) TNF-α (20 ng/mL) significantly increased cell viability of HSC-4 cells, and *IL-37* significantly alleviated TNF-α (20 ng/mL)-induced increase of cell viability of HSC-4 cells. (e) LPS (50 μg/mL) potently increased migration of HN13 cells, and IL-37 significantly alleviated TNF-α (20 ng/mL)-induced increase of cell viability of HN13 cells. (f) LPS (50 μg/mL) potently increased migration of HSC-4 cells, and IL-37 significantly alleviated TNF-α (20 ng/mL)-induced increase of cell viability of HSC-4 cells.

Enterobacterial LPS has been identified to enhance the invasion and migration of cancer cells [18]. In this study, LPS significantly increased the cell viability of HN13 (Figure 2a) and HSC-4 (Figure 2b) cells. Further investigation into the effects of IL-37 on the growth of oral cancer cells revealed that co-treatment of LPS and IL-37 resulted in a significant alleviation of the LPS-induced increase in cell viability for both HN13 and HSC-4 cells (Figure 2a and b). Additionally, TNF-α, a known promoter of cancer cell proliferation [19], was found to significantly increase the cell viability of HN13 (Figure 2c) and HSC-4 (Figure 2d) cells. Importantly, IL-37 was observed to significantly alleviate the TNF-α-induced increase in cell viability for both HN13 and HSC-4 cells (Figure 2c and d).

To further explore the impact of IL-37 on the migration of oral cancer cells, a transwell migration experiment was conducted. The results revealed that LPS (50 μg/mL) robustly increased the migration of HN13 (Figure 2e) and HSC-4 (Figure 2f) cells. Notably, IL-37 significantly alleviated the LPS-induced increase in migration for both HN13 and HSC-4 cells (Figure 2e and f). Collectively, these findings demonstrate that IL-37 effectively mitigates the proliferation and migration induced by LPS and TNF-α in oral cancer cells.

### 3.3 KD of IL-37 exacerbated LPS and TNF-α-induced proliferation of oral cancer cells

**Figure 3:**
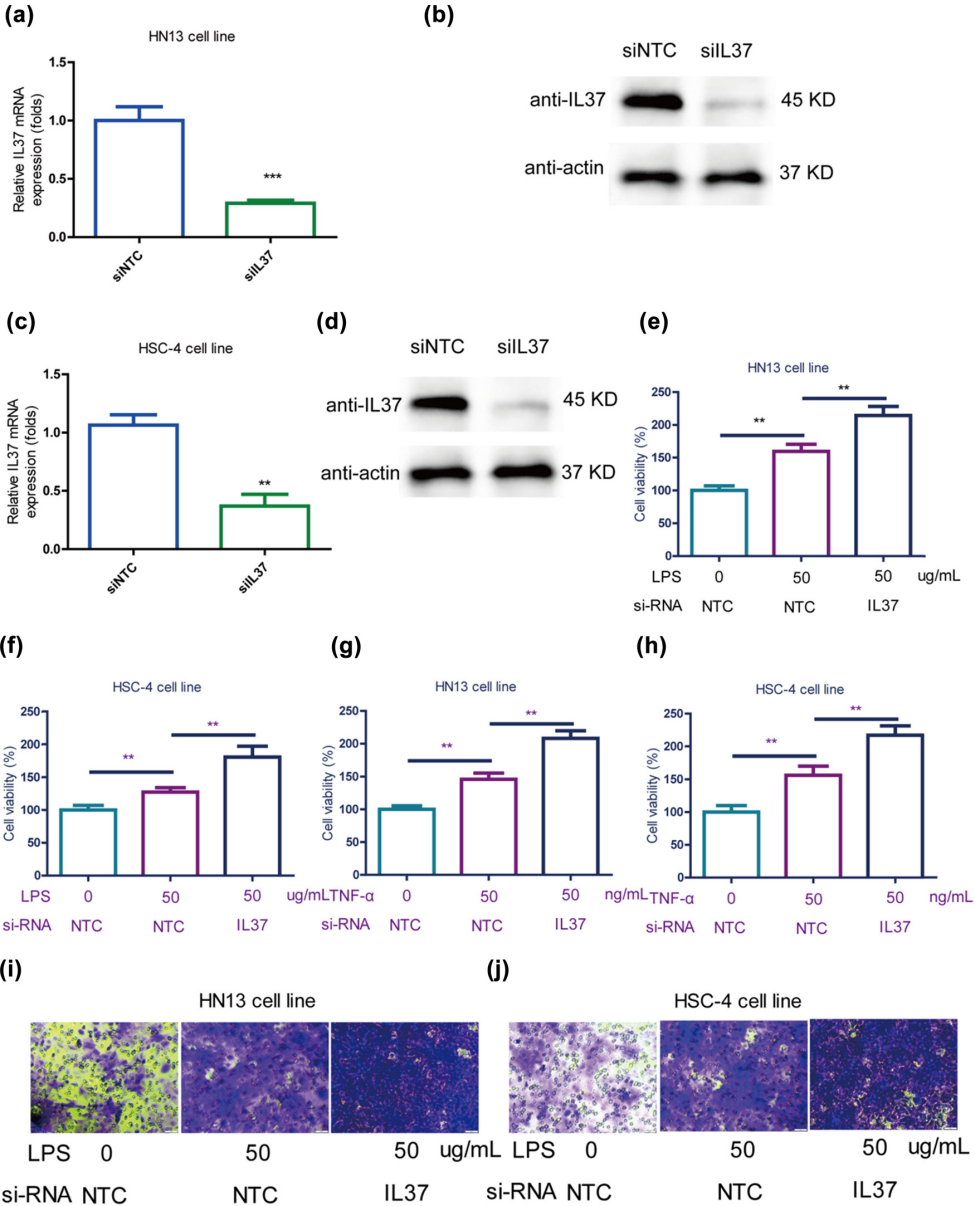
KD of *IL-37* exacerbated LPS and TNF-α-induced proliferation of oral cancer cells. (a) *IL-37* was knocked-down in HN13 cells detected by qRT-PCR. (b) *IL-37* was knocked-down in HN13 cells detected by western blot. (c) *IL-37* was knocked-down in HSC-4 cells detected by qRT-PCR. (d) *IL-37* was knocked-down in HSC-4 cells detected by western blot. (e) *IL-37* further increased LPS-induced promotion on cell viability of HN13 cells. (f) *IL-37* further increased LPS-induced promotion on cell viability of HSC-4 cells. (g) *L-37* further increased TNF-α (20 ng/mL)-induced promotion on cell viability of HN13 cells. (h) L-37 further increased TNF-α (20 ng/mL)-induced promotion on cell viability of HSC-4 cells. (i) *IL-37* KD further increased TNF-α (20 ng/mL)-induced promotion on migration of HN13 cells. (j) *IL-37* KD further increased TNF-α (20 ng/mL)-induced promotion on migration of HSC-4 cells.

To further investigate the impact of IL-37 on oral cancer cells, the study explored the effects of *IL-37* KD on the growth of oral cancer cells. First of all, IL-37 KD was confirmed by qRT-PCR (Figure 3a) and western blot (Figure 3b) in HN13 cells. Similarly, *IL-37* KD was confirmed by qRT-PCR (Figure 3c) and western blot (Figure 3d) in HSC-4 cells. The results indicated that *IL-37* KD further increased the LPS-induced promotion of cell viability in HN13 cells (Figure 3e) and HSC-4 cells (Figure 3f). Similarly, it was found that *IL-37* KD further increased the *TNF-α* (20 ng/mL)-induced promotion of cell viability in HN13 cells (Figure 3g) and HSC-4 cells (Figure 3h).

Additionally, to verify the effects of *IL-37* on the migration of oral cancer cells, a transwell migration experiment was conducted. The results showed that IL-37 KD further increased the TNF-α (20 ng/mL)-induced promotion of migration in both HN13 (Figure 3i) and HSC-4 (Figure 3j) cells. Collectively, these findings demonstrate that KD of IL-37 exacerbates the LPS- and TNF-α-induced proliferation and migration of oral cancer cells.

### 3.4 IL-37 inhibited inflammation on oral cancer cells

**Figure 4:**
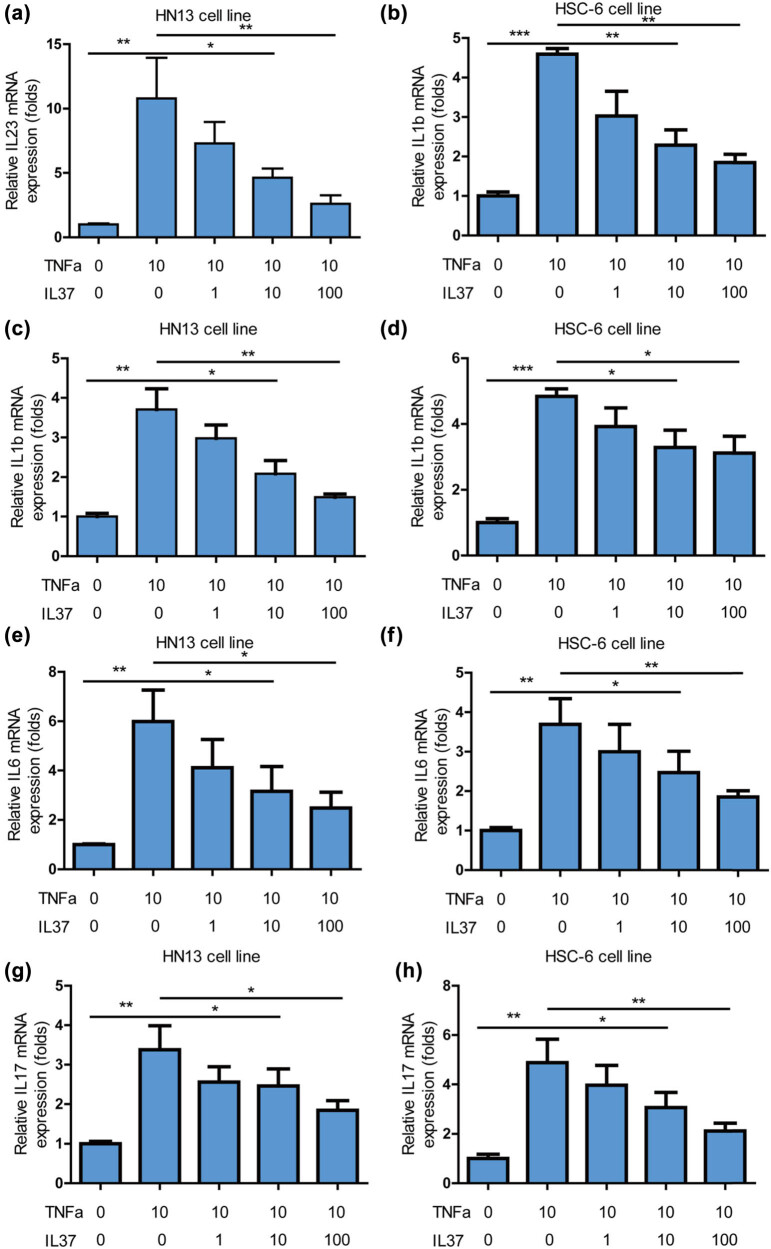
*IL-37* inhibited inflammation on oral cancer cells. (a) *IL-37* potently inhibited TNF-α-induced increase of pro-inflammation gene *IL23* on HN13 cells. (b) *IL-37* potently inhibited TNF-α-induced increase of pro-inflammation gene IL23 on HSC-6 cells. (c) *IL-37* potently inhibited TNF-α-induced increase of pro-inflammation gene IL1B on HN13 cells. (d) *IL-37* potently inhibited TNF-α-induced increase of pro-inflammation gene *IL1B* on HSC-6 cells. (e) *IL-37* potently inhibited TNF-α-induced increase of pro-inflammation gene *IL6* on HN13 cells. (f) *IL-37 p*otently inhibited TNF-α-induced increase of pro-inflammation gene IL6 on HSC-6 cells. (g) *IL-37* potently inhibited TNF-α-induced increase of pro-inflammation gene *IL17* on HN13 cells. (h) *IL-37* potently inhibited TNF-α-induced increase of pro-inflammation gene *IL17* on HSC-6 cells.

The increasing body of evidence suggests that IL-37 possesses potent anti-inflammatory effects [20]. This prompted an investigation into the impact of IL-37 on inflammation in oral cancer cells. The study revealed that IL-37 robustly inhibited TNF-α-induced upregulation of the pro-inflammatory gene *IL23* in HN13 (Figure 4a) and HSC-6 (Figure 4b) cells. IL-37 also significantly suppressed TNF-α-induced elevation of the pro-inflammatory gene IL1B in HN13 (Figure 4c) and HSC-6 (Figure 4d) cells. Likewise, IL-37 effectively curtailed TNF-α-induced increase in the pro-inflammatory gene *IL6* in HN13 (Figure 4e) and HSC-6 (Figure 4f) cells. In parallel, IL-37 demonstrated a potent inhibitory effect on TNF-α-induced upregulation of the pro-inflammatory gene *IL17* in HN13 (Figure 4g) and HSC-6 (Figure 4h) cells. In summary, these findings indicate that IL-37 inhibits inflammation in oral cancer cells.

### 3.5 STAT3 closely regulated inflammation on oral cancer cells

**Figure 5:**
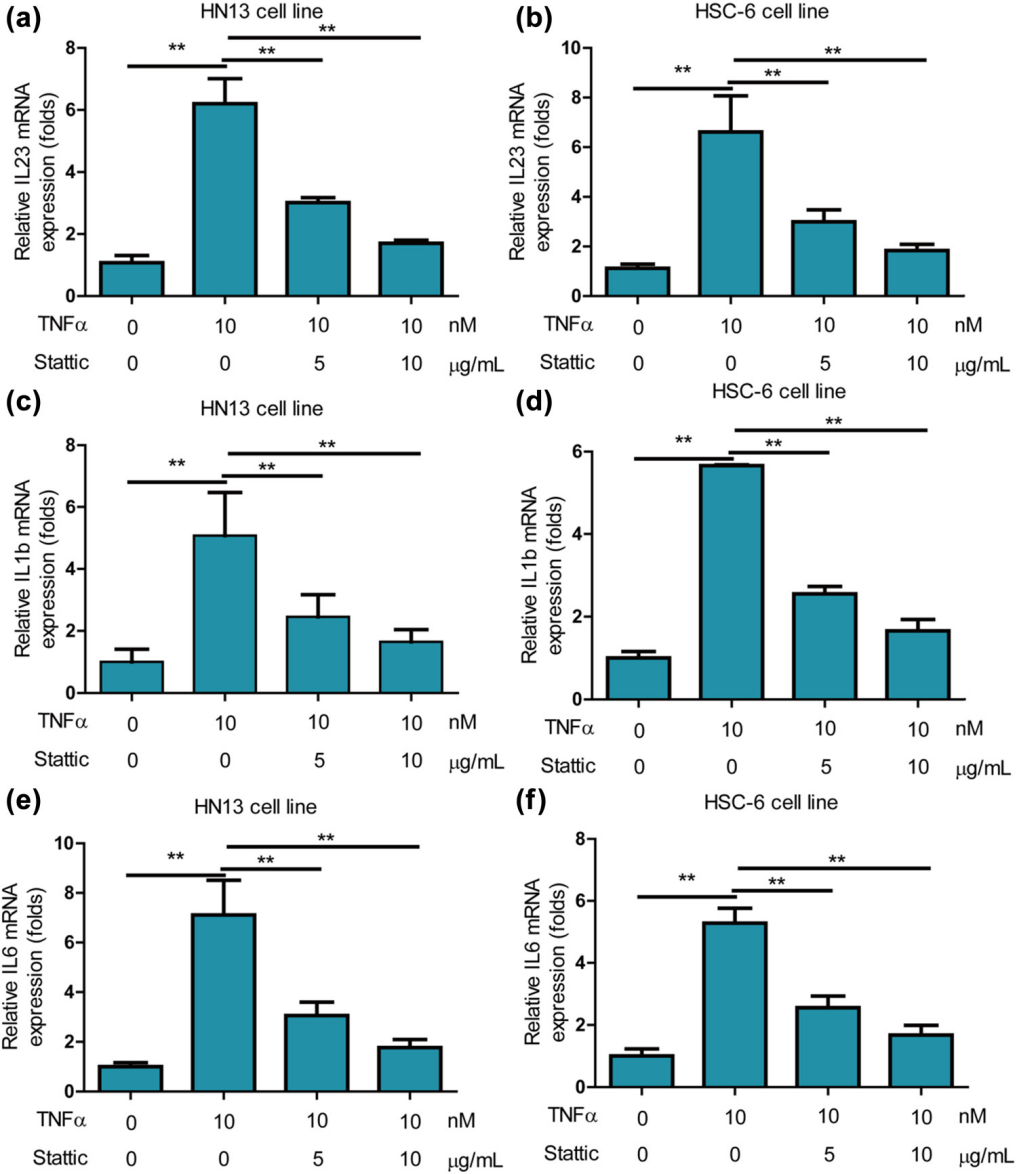
STAT3 closely regulated inflammation on oral cancer cells. (a) The STAT3 inhibitor stattic potently alleviated promotion effects of TNF-α (10 ng/mL) on inflammation gene *IL23* on HN13 cells. (b) Stattic potently alleviated promotion effects of TNF-α (10 ng/mL) on inflammation gene *IL23* on HSC-6 cells. (c) Stattic potently alleviated promotion effects of TNF-α (10 ng/mL) on inflammation gene *IL1b* on HN13 cells. (d) Stattic potently alleviated promotion effects of TNF-α (10 ng/mL) on inflammation gene *IL23* on HSC-6 cells. (e) Stattic potently alleviated promotion effects of TNF-α (10 ng/mL) on inflammation gene *IL6* on HN13 cells. (f) Stattic potently alleviated promotion effects of TNF-α (10 ng/mL) on inflammation gene *IL6* on HSC-6 cells.

*STAT3* has been reported to play a critical role in the regulation of inflammation and the growth of cancer cells [21]. Consequently, the study explored the effects of *STAT3* on inflammation in oral cancer cells. It was observed that the STAT3 inhibitor, stattic, robustly alleviated the promotional effects of TNF-α (10 ng/mL) on the inflammation gene *IL23* in HN13 cells (Figure 5a) and HSC-6 cells (Figure 5b). Furthermore, stattic significantly mitigated the promotional effects of TNF-α (10 ng/mL) on the inflammation gene IL1b in HN13 cells (Figure 5c) and HSC-6 cells (Figure 5d). Simultaneously, stattic effectively attenuated the promotional effects of TNF-α (10 ng/mL) on the inflammation gene *IL6* in HN13 cells (Figure 5e) and HSC-6 cells (Figure 5f). These findings collectively demonstrate that STAT3 closely regulates inflammation in oral cancer cells.

### 3.6 Stattic significantly compromised inhibitory effects of IL-37 on growth of oral cancer cells and promotion effects of IL-37 on apoptosis of oral cancer cells

**Figure 6:**
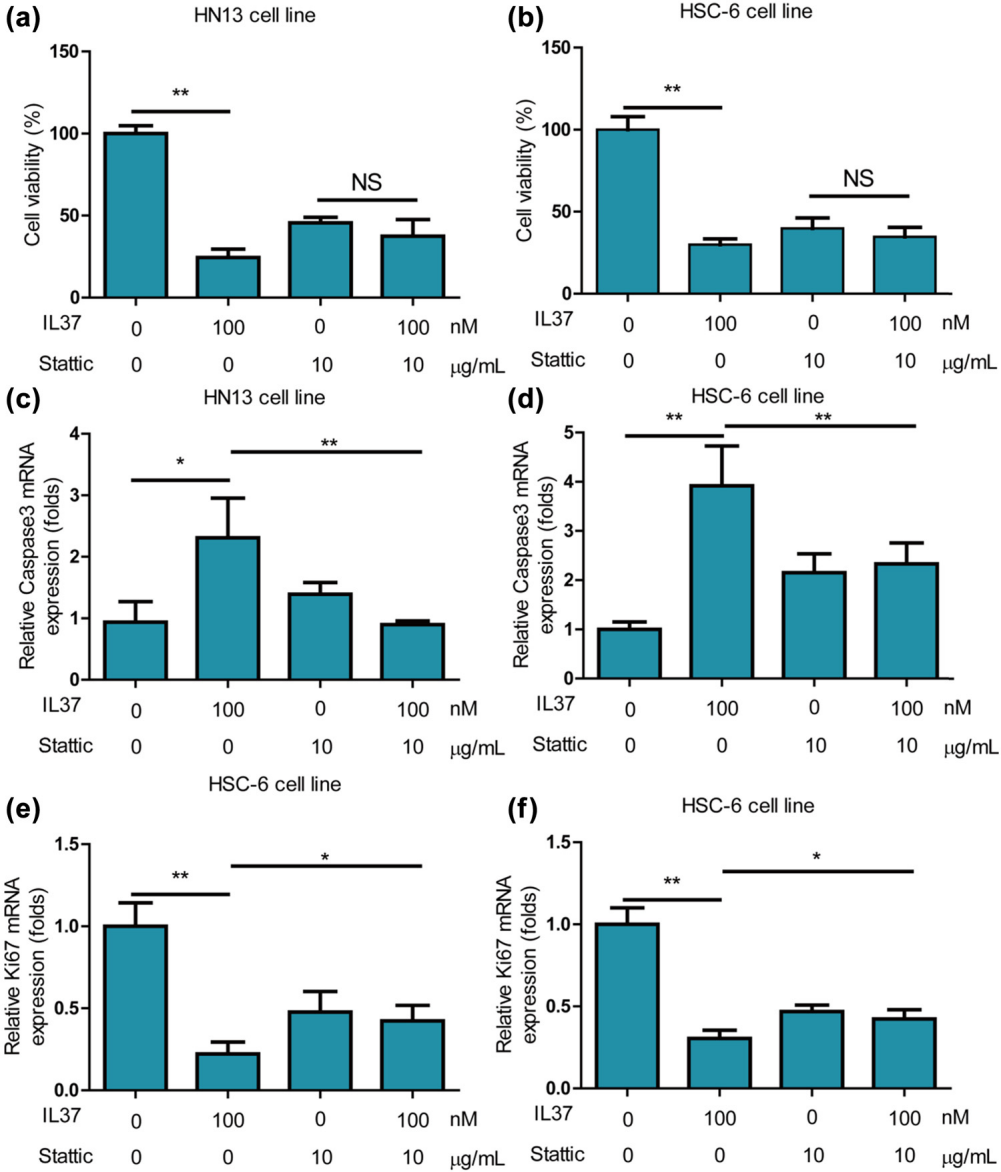
Stattic significantly compromised inhibitory effects of IL-37 on growth of oral cancer cells and promotion effects of IL-37 on apoptosis of oral cancer cells. (a) *IL-37* did not inhibit cell viability of HN13 cells under treatment of stattic. (b) *IL-37* did not inhibit cell viability of HSC-6 cells under treatment of stattic. (c) *IL-37* did not increase cell apoptosis of HN13 cells under treatment of stattic. (d) *IL-37* did not increase cell apoptosis of HSC-6 cells under treatment of stattic. (e) *IL-37* did not inhibit expression of proliferation gene KI67 on HN13 cells under treatment of stattic. (f) *IL-37* did not inhibit expression of proliferation gene KI67 on HSC-6 cells under treatment of stattic.

In further investigating the mechanism by which IL-37 regulates the activities of oral cancer, oral cancer cells were co-treated with IL-37 and stattic. The results revealed that IL-37 did not inhibit the cell viability of HN13 cells under the treatment of stattic (Figure 6a). Similarly, IL-37 did not inhibit the cell viability of HSC-6 cells under the treatment of stattic (Figure 6b). Additionally, IL-37 did not increase cell apoptosis of HN13 cells (Figure 6c) and HSC-6 cells (Figure 6d) under the treatment of stattic. Furthermore, it was found that IL-37 did not inhibit the expression of the proliferation gene KI67 in HN13 cells (Figure 6e) and HSC-6 cells (Figure 6f) under the treatment of stattic. Collectively, these findings demonstrate that IL-37 regulates the growth, proliferation, and apoptosis of oral cancer cells via *STAT3*.

## 4 Discussion

The oral cavity represents the most common anatomical subsite for upper aero-digestive tract malignancies [2]. A comprehensive understanding of the pathogenesis is crucial for developing innovative therapies to treat oral cancers. In this study, IL-37 was observed to significantly reduce the cell viability of oral cancer cells and induce apoptosis. Furthermore, IL-37 mitigated the proliferation of oral cancer cells induced by LPS and TNF-α, while the KD of IL-37 exacerbated this proliferation. IL-37 was also identified to have an inhibitory effect on inflammation in oral cancer cells. Mechanistically, it was revealed that STAT3 closely regulated inflammation in oral cancer cells, and the use of stattic significantly compromised the inhibitory effects of IL-37 on the growth of oral cancer cells and the promotive effects on apoptosis.

Cytokines have been recognized as important factors to influence inflammation and cancer growth. Among cytokines, IL-37 has anti-inflammatory characteristics in both innate and acquired immune responses by downregulating pro-inflammatory molecules, which is quite different from other cytokines including *IL6*, *IL11*, *IL22*, and IL31 that exert pro-inflammatory effects [22]. IL-37 was initially identified *in silico* in 2000 [23]. Since then, as a member of the IL-1 family, it has been recognized for its diverse life and cellular activities, encompassing inflammation and tumor regulation [23]. IL-37 comprises five variants (a, b, c, d, and e) and serves as both an intracellular and an extracellular cytokine [23]. Studies have highlighted the significance of IL-37 in various cancer contexts. For instance, in breast cancer, circulating IL-37 expression was reported to be highest in ER+/PR+/HER2+ patients compared to PR+ breast cancer patients, emphasizing a potential role in prognosis regulation through ER+/PR+/HER2+ signaling [24]. Additionally, *IL-37* expression correlated with serum alpha-fetoprotein and tumor size in HCC and paracancerous tissues, showing a negative correlation with NF-κB protein expression in HCC tissues and liver cancer cell lines [25]. IL-37, when combined with radiation therapy (RT), enhanced RT-induced inhibition of cell proliferation and apoptosis in prostate cancer cells [26]. In gallbladder cancer, IL-37 was reported to suppress migration and invasion by inhibiting HIF-1α-induced epithelial–mesenchymal transition [27]. Furthermore, IL-37 was found to inhibit invasion in human cervical cancer cells via suppression of runt-related transcription factor 2 [28]. In the present study, IL-37 demonstrated significant effects on oral cancer cells, including a decrease in cell viability and an increase in apoptosis (Figure 1), while IL-37 has minor effect of growth normal oral cell line (Figure S3). Moreover, IL-37 mitigated LPS and TNF-α-induced proliferation and migration of oral cancer cells (Figure 2). This aligns with findings in gallbladder cancer where IL-37 suppressed migration and invasion [27]. The KD of IL-37 further exacerbated LPS and TNF-α-induced proliferation of oral cancer cells (Figure 3), suggesting that IL-37 may play a crucial role in the activities of various cancer types, including oral cancer.

Accumulating evidence suggests a close association between inflammation and the development and progression of cancer [29]. Inflammation, considered a fundamental innate immune response to tissue perturbations, exerts its influence throughout various stages of tumor development and treatment [29]. Cytokines play a crucial role in mediating cell communication and coordinating complex multicellular behaviors, contributing significantly to inflammation [30]. A substantial body of literature supports the idea that mature IL-37, following activating cleavage by caspase-1, translocates to the nucleus, where it suppresses the transcription of pro-inflammatory genes [20]. This study observed that IL-37 exhibited potent inhibitory effects on the TNF-α-induced expression of inflammation-related genes, including *IL23*, *IL1b*, *IL6*, and *IL17* in oral cancer cells (Figure 4). This aligns with previous studies demonstrating that *IL-37b* can inhibit the *in vitro* induction of pro-inflammatory cytokines (*IL-6* and *TNF-α*) and chemokines (*CXCL8*, *CCL2*, and *CCL5*) related to atopic dermatitis. Moreover, IL-37 was found to modulate autophagosome biogenesis-related *LC3B* and decrease autophagy-associated ubiquitinated protein *p62* by regulating intracellular AMP-activated protein kinase and *mTOR* signaling pathways [31]. These findings underscore the crucial role of IL-37 in regulating inflammation and the pathogenesis of tumors, including oral cancer.

Recent evidence underscores the pivotal role of *STAT* family proteins, particularly *STAT3*, in selectively inducing and maintaining a pro-carcinogenic inflammatory microenvironment during malignant transformation and cancer progression [31]. *STAT3* has been reported to control the ability of pre-neoplastic and malignant cells to resist apoptosis-based tumor-surveillance, regulate tumor angiogenesis, and promote invasiveness [31]. The link between *STAT3* and inflammation-associated tumorigenesis is established, often initiated by genetic alterations in malignant cells [31]. In the realm of immunity and inflammation, autosomal dominant *STAT3* inactivating mutations associated with hyper immunoglobulin E syndrome highlight the causal role of *STAT3* loss-of-function in human immune diseases [32]. This study observed that the *STAT3* inhibitor stattic robustly compromised TNF-α-induced upregulation of inflammation-related genes in oral cancer cells (Figure 5), emphasizing the significant role of *IL-37* in modulating inflammation in these cells. Previous literature has shown that *IL-37* inhibits invasion and metastasis in non-small cell lung cancer by suppressing the *IL-6*/*STAT3* signaling pathway [33]. Similarly, this study found that the *STAT3* inhibitor stattic further promoted LPS- and TNF-α-induced increases in cell viability and migration of oral cancer cells (Figure 6), highlighting the crucial role of *IL-37* in regulating the growth, proliferation, and migration of oral cancer cells.

## 5 Limitations and future direction

Although the present study has comprehensively demonstrated that *IL-37* harbors anti-inflammatory effects on oral cancer cells, which is through the *JAK2*/*STAT3* signaling pathway by using two different types of cell models. Several limitations exist in the study, which should be solved in the future study. Cell lines may not fully encompass the heterogeneity of oral cancer in patients. Therefore, animal models are planned to be used in the future study. In the study, a commonly used migration assay, transwell migration assay was used to evaluate migration of oral cancer cells. While more migration assays have been reported and established including scratch assays, microfluidic chamber assays, and so on [34]. Thus, more migration assays are planned to be used in the future study. Since this study has found that IL-37 plays an important role in oral cancer cell growth, while it is attractive to investigate effects of IL-37 on other types of cancer cells such as colon cancer, liver cancer, and so on.

## 6 Conclusion

In summary, this study delved into the impact of *IL-37* on inflammation, proliferation, apoptosis, and migration in oral cancer cells. The results revealed that *IL-37* significantly curtailed cell viability while promoting apoptosis in oral cancer cells. Moreover, *IL-37* mitigated the proliferation induced by LPS and *TNF-α*, and the KD of *IL-37* exacerbated the proliferation triggered by LPS and TNF-α in oral cancer cells. *IL-37* demonstrated a suppressive effect on inflammation in oral cancer cells. The modulatory effects of *IL-37* on inflammation, proliferation, apoptosis, and migration were found to be mediated through *STAT3*. These findings are anticipated to offer valuable insights into a deeper understanding of the pathogenesis of oral cancers and contribute to the development of novel drugs for treating this disease.

## Supplementary Material

Supplementary Figure
