# Supplementary material for: Interleukin-37 mediates the anti-oral tumor activity in oral cancer through STAT3
Source: Open Med (Wars). 2025 May 26;20(1):20251173. doi: 10.1515/med-2025-1173 (PMC12120405; doi:10.1515/med-2025-1173)
Supplement: Supplementary Figure [file med-2025-1173-sm.pdf]

Supplementary material

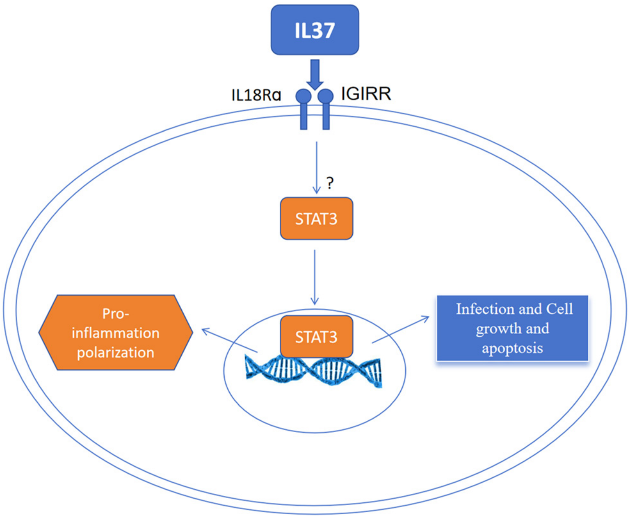

Figure S1: Mode-of-action of IL-37 involving in inflammation, infection, and cell growth and apoptosis.

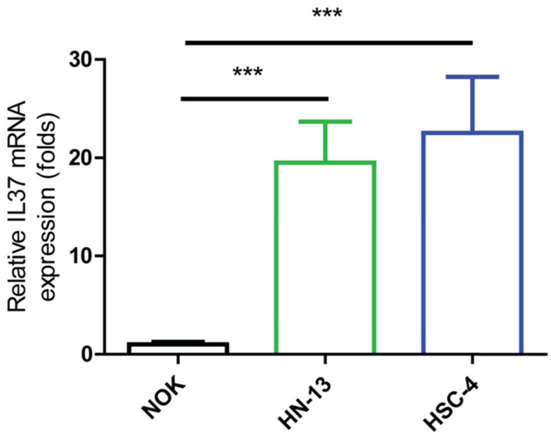

Figure S2: expression level of IL-37 in NO (average CT is 27.52366132), HN-13 (average CT is 23.14847493) and HSC-4 (average CT is 22.98979849) cell lines.

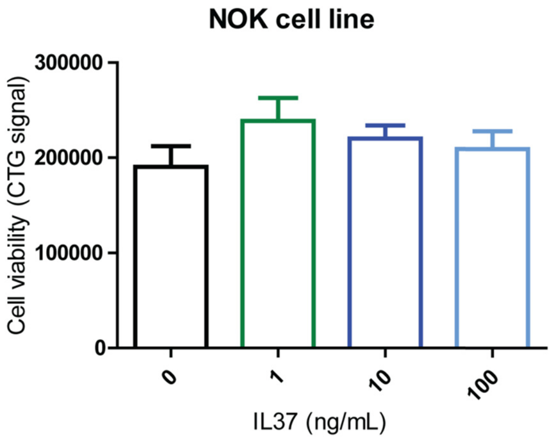

Figure S3: Effect of IL-37 on growth of NOK cell line.
